# Polymer-Induced Swelling of Solid-Supported Lipid Membranes

**DOI:** 10.3390/membranes6010002

**Published:** 2015-12-23

**Authors:** Martin Kreuzer, Marcus Trapp, Reiner Dahint, Roland Steitz

**Affiliations:** 1 Helmholtz-Zentrum Berlin für Materialien und Energie GmbH, Hahn-Meitner-Platz 1, Berlin 14109, Germany; marcus.trapp@helmholtz-berlin.de; 2 Angewandte Physikalische Chemie, Ruprecht-Karls-Universität Heidelberg, Im Neuenheimer Feld 253, Heidelberg 69120, Germany; reiner.dahint@pci.uni-heidelberg.de

**Keywords:** lipid, membranes, polymer, neutron reflectivity

## Abstract

In this paper, we study the interaction of charged polymers with solid-supported 1,2-dimyristoyl-sn-glycero-3-phosphocholine (DMPC) membranes by *in-situ* neutron reflectivity. We observe an enormous swelling of the oligolamellar lipid bilayer stacks after incubation in solutions of poly(allylamine hydrochloride) (PAH) in D_2_O. The positively charged polyelectrolyte molecules interact with the lipid bilayers and induce a drastic increase in their *d*-spacing by a factor of ~4. Temperature, time, and pH influence the swollen interfacial lipid linings. From our study, we conclude that electrostatic interactions introduced by the adsorbed PAH are the main cause for the drastic swelling of the lipid coatings. The DMPC membrane stacks do not detach from their solid support at *T* > *T_m_*. Steric interactions, also introduced by the PAH molecules, are held responsible for the stabilizing effect. We believe that this novel system offers great potential for fundamental studies of biomembrane properties, keeping the membrane’s natural fluidity and freedom, decoupled from a solid support at physiological conditions.

## 1. Introduction

Biological cell membranes are selectively permeable for macromolecules and are constantly interacting with their surroundings. The membranes are composed of lipids, proteins and other macromolecules [1]. For an improved understanding of this vital component of organisms, it is relevant to study the membranes’ interaction with various molecules. The interplay of natural occurring and synthetic polymers with biological membranes is of great interest in the medical field, e.g., in drug delivery [2] and nanotechnology therapeutics [3,4], where for instance positively charged polymer-based nanoparticle (NP) constructs are used in nonviral gene transfection [5]. Wang and coworkers have utilized covalently assembled PAH/glutaraldehyde microcapsules and demonstrated their easy ingestion by human cells via endocytosis pathways [6]. NP uptake starts with the contact of NP and cell membrane. On the molecular level, it is initiated by the interaction of a PAH chain with the lipid headgroups of the outer bilayer leaflet. This intimate contact and adsorption of the polyelectrolyte to the lipid membrane can imply profound changes of the functionality of the membrane system [7]. Using lipid vesicles as a model for biological membranes, the affinity of polyelectrolytes to adsorb to the outer membranes was shown by Quemeneur and coworkers [8,9]. The authors used chitosan and hyaluronic acid (HA) as natural occurring molecules of the extra-cellular matrix and investigated their interactions with zwitterionic lipid membranes of vesicles [8]. In this context, a numerical study revealed that physiological conditions with a Debye length *k*^−1^ ~ 10 Å represent the optimum condition for wrapping a sphere of the order of a unilamellar vesicle (*R_s_* = 50 Å) at minimum surface charge by a semiflexible polymer chain of intermediate length (*L* = 500 Å) [10]. On the micrometer scale, fluorescent microscopy observations showed that the lipid vesicles were decorated by the polymers in a broad range of solution pH [9]. The inverse situation was also investigated: Deposition of zwitterionic lipid membranes by vesicle fusion was possible on negatively charged polymer surfaces, while the lipid membranes did not adsorb on a positively charged polymer coating [11]. Singh *et al.* demonstrated the deposition of a free floating DPPC lipid bilayer on a positively charged polyelectrolyte cushion and detected structural differences as a function of the pH of the surrounding solution [12]. These results reveal a strong influence of the free charges in the system. Recent experiments further showed that a substrate-bound lipid coating drastically changes in structure upon incubation in a solution of a negatively charged polysaccharide in D_2_O [13]. The *d*-spacing of the lipid coating increased from 65 Å in D_2_O to 247 Å in the presence of HA in D_2_O. Besides the large swelling the coating did not detach and remained stable on the substrate, even in the liquid-like state of the DMPC membranes at 39 °C. The observed stability above the main phase transition does not occur in the pure DMPC/D_2_O system [13,14].

The present study is focused on the interaction of a synthetic polyelectrolyte with DMPC lipid coatings. Two different temperatures were chosen for the experiments: 20 °C, *i.e.*, *T* < *T_m_*, where *T_m_* ≈ 24 °C is the main phase transition temperature of the lyotropic bulk system [15], and 38 °C, *i.e.*, *T* > *T_m_*. In order to study the influence of the molecular weight of the polymer on the lipid membranes, the experiments were performed with two different types of PAH, namely PAH-15, *M_w_* = 15 kDa, and PAH-58, *M_w_* = 58 kDa, respectively. Furthermore, the pH of the solution was changed so as to study the influence of the electrostatic forces within the system. The premise that charged polymers have a profound impact on the morphology and the structure of the supported lipid membrane systems was investigated *in-situ* by neutron reflectivity.

## 2. Results and Discussion

### 2.1. Dry State

**Figure 1:**
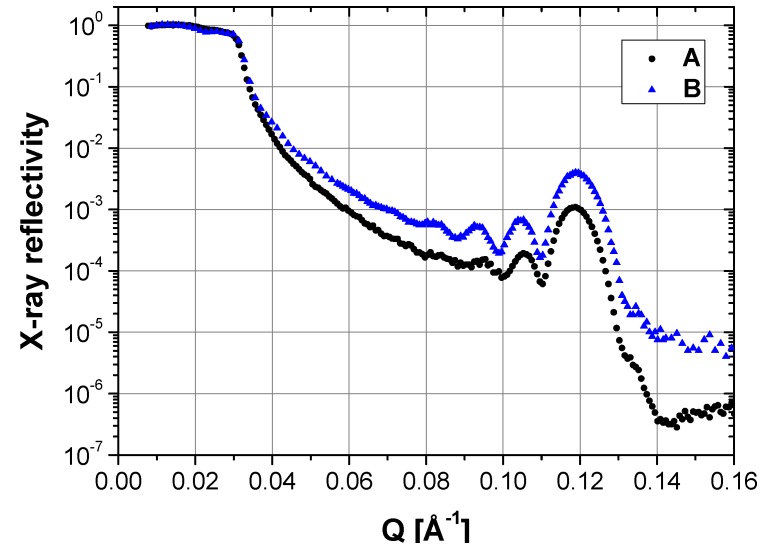
X-ray reflectivity of the DMPC coatings A and B at room temperature and ambient relative humidity in air, prior to incubation in PAH solutions.

Identically prepared DMPC coatings, labeled A and B, were produced on two identical silicon substrates by the protocol introduced by Mennicke and Salditt [16]. The X-ray reflectivity (XRR) measurements conducted on the as-prepared samples at room temperature and ambient relative humidity in air are depicted in Figure 1. The reflectivity profiles show well-pronounced Kiessig fringes and a first order Bragg peak at *Q* = 0.12 Å^−1^. For both samples, the *d*-spacing of *d* = 52.7 ± 0.3 Å, as determined from the Bragg peak, is in good agreement with the literature for partially hydrated DMPC lipid membranes in $P_{ß}^{\prime }$ state [16]. Besides the homogeneity of the samples, reflected in the height of the peaks, the total thickness of the oligolamellar lipid bilayer stacks - calculated from the maxima positions of the Kiessig fringes - was *t* = 505 ± 10 Å with a number of *N* = *t*/*d* = 10 ± 1 lipid membranes/stack in both cases.

### 2.2. Wet State

**Figure 2:**
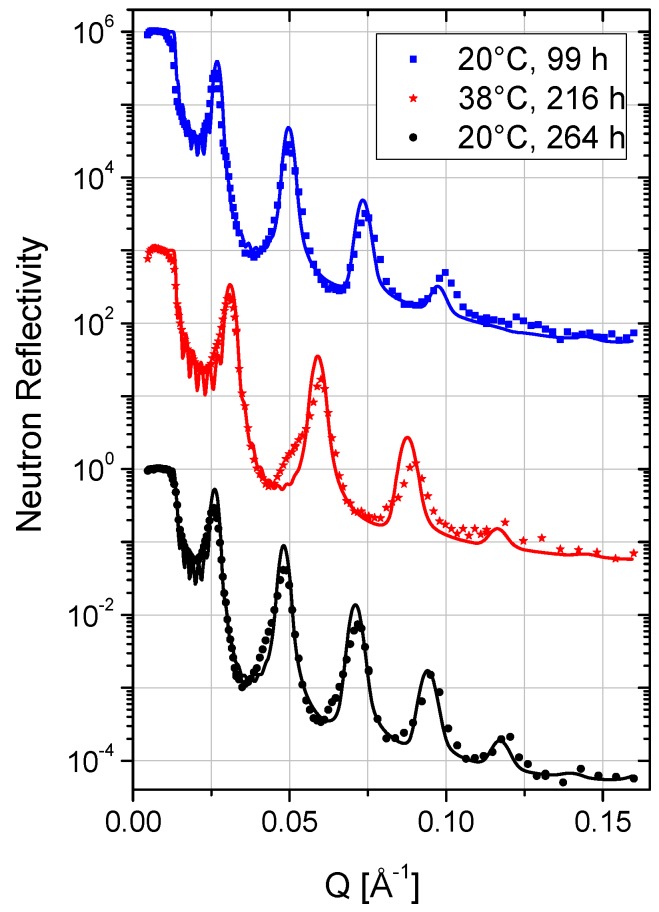
Neutron reflectivity curves of sample A, incubated in a solution of PAH-58 in D_2_O at pH = 5 at 20 °C, 38 °C and again at 20 °C from top to bottom. For clarity, the curves are shifted vertically. Solid lines are best fits to the data according to the model gathered in Table 1.

**Figure 3:**
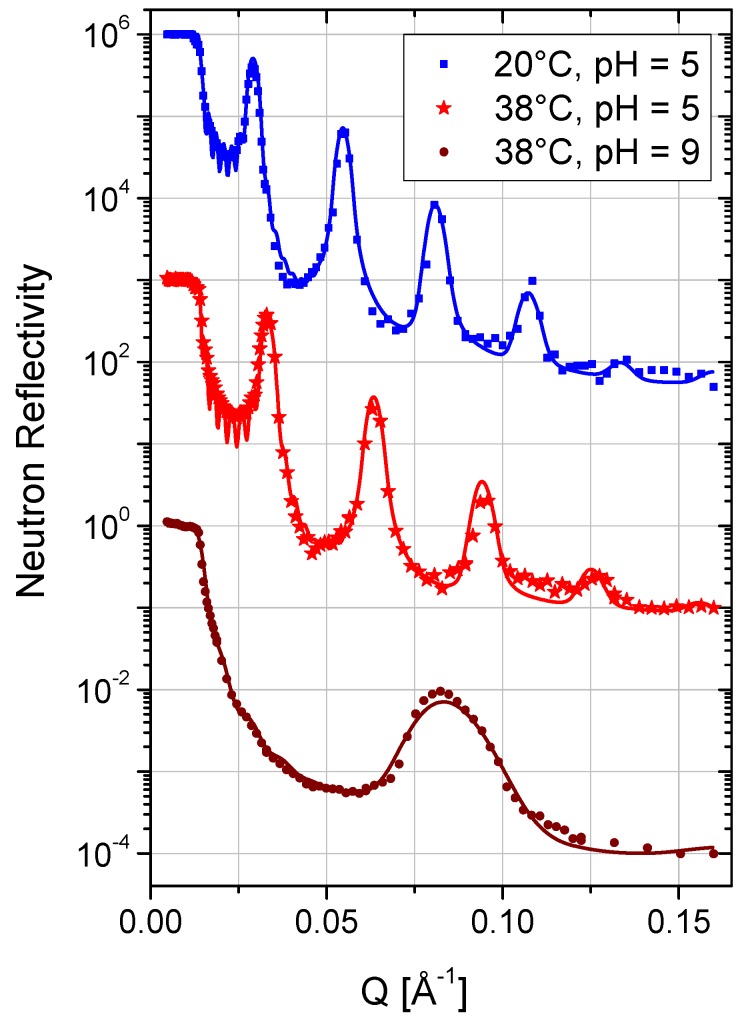
Neutron reflectivity curves of sample B, incubated in a solution of PAH-15 in D_2_O at pH = 5 at 20 °C and 38 °C, and at pH = 9 at 38 °C. For clarity, the curves are shifted vertically. Solid lines are best fits to the data according to the model gathered in Table 2.

The solid-supported lipid membrane stacks were incubated at 20 °C in a solution of PAH-58 in case of sample A and in a solution of PAH-15 in case of sample B, respectively, and characterized *in-situ* at the liquid–solid interface by neutron reflectometry. Figure 2 shows the neutron reflectivity of sample A, measured 99 h (*i.e.*, about four days) after incubation in a solution of 3 mg/mL PAH-58 in D_2_O. The measurement unravels a highly ordered, oligolamellar coating with a drastically increased *d*-spacing. Up to four Bragg peaks are clearly visible, originating from the set of bilayer membranes aligned parallel to the interface. The Bragg peak analysis revealed a *d*-spacing of *d* = 257 ± 2 Å. Follow-up measurements, 125 h and 133 h after incubation, proved equilibrium conditions, *i.e.*, no further variation in *d*-spacing. Sample B was incubated with a solution of 3 mg/mL PAH-15 in D_2_O in order to investigate the potential influence of the molecular weight on the swelling of the samples. Figure 3 displays the neutron reflectivity of sample B, measured 248 h after incubation. The peak analysis revealed a *d*-spacing of *d* = 239 ± 1 Å at 20 °C in that case.

**Table 1:** Structural parameters of sample A incubated with PAH-58 in D_2_O (3 mg/mL).

Layer			20 °C			38 °C			20 °C		
		*No.*	*d* (Å)	*SLD* (10^−6^ Å^−2^)	ϕ_*sol*_ (%)	*d* (Å)	*SLD* (10^−6^ Å^−2^)	ϕ_*sol*_ (%)	*d* (Å)	*SLD* (10^−6^ Å^−2^)	ϕ_*sol*_ (%)
Si		–	s-i	2.07	–	s-i	2.07	–	s-i	2.07	–
SiO_2_		1	13.0	3.47	0	13.0	3.47	0	13.0	3.47	0
Heads_a_		2	11.5	3.44	*0*	8.8	3.44	*0*	11.5	3.44	*0*
Tails_a_		3	30.3	−0.2	**0 ± 2**	25.3	−0.2	**0 ± 6**	30.3	−0.2	**0 ± 5**
Heads_a_		2	11.5	3.44	*0*	8.8	3.44	*0*	11.5	3.44	*0*
PAH		4	*9.5*	0	*60*	*12.4*	*0*	*60*	*7.5*	*0*	*60*
9 ×	(Interlayer	5	**188.3 ± 2.9**	5.7	–	**149.7 ± 2.4**	5.7	–	**201.0 ± 2.6**	5.7	–
	PAH ^§^	4	**9.5 ± 2.8**	0	**60 ± 9**	**12.4 ± 2.4**	0	**60 ± 9**	**7.5 ± 2.5**	0	**60 ± 8**
	Heads ^$^	6	11.5	3.44	*45*	8.8	3.44	*29*	11.5	3.44	*22*
	Tails ^&^	7	30.3	−0.2	**45 ± 2**	25.3	−0.2	**29±3**	30.3	−0.2	**22 ± 2**
	Heads	6	11.5	3.44	*45*	8.8	3.44	*29*	11.5	3.44	*22*
	PAH)	4	*9.5*	0	*60*	*12.4*	0	*60*	*7.5*	0	*60*
D_2_O ^£^		–	s-i	5.7	–	s-i	5.7	–	s-i	5.7	–

Notes: Bold parameters were varied during fitting, parameters in *italic* were dependent on values of related bold parameters, and all other parameters were fixed. ^§^ *SLD_PAH_* was calculated on basis of the molecular composition, the volume of the monomer unit, *V_AH_* = 67 Å^3^ [17] and the sum of the respective, tabulated atomic scattering lengths, (∑ *b*)_*AH*_ [18]. ^$^ *SLD_Heads_* was determined from the molecular volume of the DMPC headgroup, *V_h_* = 319 Å^3^, retrieved from Petrache and coworkers [19] and 7.3 water molecules, *n_w_*, associated with each headgroup. *SLD_Heads_* = (∑ *b*)_*h*_ + *n_w_* ⋅ (∑ *b*)_*w*_/(*V_h_* + *n_w_* ⋅ *V_w_*) with the volume of a water molecule *V_w_* = 30 Å^3^. ^&^ The thickness of the aliphatic tails slab, *d_Tails_*, was calculated by linear extrapolation to 38 °C from measured values at 30 °C and 50 °C [20]. *SLD_Tails_* = (∑ *b*)_*t*_/(*A*(*T*) ⋅ *d_Tails_*/2) with $A\left(T\right)=A_{0}(1+\frac{1}{A_{0}}\left(\frac{dA}{dT}\right)\cdot \left(T-T_{0}\right)$. *A*_0_ = 59.9 Å^2^ (30 °C) and $\frac{1}{A_{0}}\left(\frac{dA}{dT}\right)$ = 0.0032 K^−1^, T = 38 °C, T_0_ = 30 °C. [20]. ^£^ The SLD_D2O_ deviated from its literature value due to contamination with H_2_O at a volume fraction of 0.095. The change in SLD due to solvated PAH at a concentration of 3 mg PAH/mL D_2_O is negligible; “s-i” = semi-infinite. Scattering background was set to 5 × 10^−5^, Δ*Q*/*Q* was set to 5%.

**Table 2:** Structural parameters of sample B incubated with PAH-15 in D_2_O (3 mg/mL).

Layer			20 °C, pH = 5			38 °C, pH = 5			38 °C, pH = 9		
		*No.*	*d* (Å)	*SLD* (10^−6^ Å^−2^)	ϕ_*sol*_ (%)	*d* (Å)	*SLD* (10^−6^ Å^−2^)	ϕ_*sol*_ (%)	*d* (Å)	*SLD* (10^−6^ Å^−2^)	ϕ_*sol*_ (%)
Si		–	s-i	2.07	–	s-i	2.07	–	s-i	2.07	–
SiO_2_		1	13.0	3.47	0	13.0	3.47	0	13.0	3.47	0
Heads_a_		2	11.5	3.56	*0*	8.8	3.56	*0*	8.8	3.56	*0*
Tails_a_		3	30.3	−0.2	**0 ± 3**	25.3	−0.2	**0 ± 6**	25.3	−0.2	**0 ± 4**
Heads_a_		2	11.5	3.56	*0*	8.8	3.56	*0*	8.8	3.56	*0*
PAH		4	*8.7*	0	*65*	*11.0*	*0*	*65*	*12.0*	*0*	*70*
9 ×	(Interlayer	5	**165.5 ± 2.6**	6.0	–	**137.1 ± 2.3**	6.0	–	**9.2 ± 1.0**	6.0	–
	PAH ^§^	4	**8.7 ± 2.5**	0	**65 ± 4**	**11.0 ± 2.2**	0	**65 ± 4**	**12.0 ± 1.0**	0	**70 ± 4**
	Heads ^$^	6	11.5	3.56	*21*	8.8	3.56	*21*	8.8	3.56	*17*
	Tails ^&^	7	30.3	−0.2	**21 ± 2**	25.3	−0.2	**21 ± 2**	25.3	−0.2	**17 ± 2**
	Heads	6	11.5	3.56	*21*	8.8	3.56	*21*	8.8	3.56	*17*
	PAH)	4	*8.7*	0	*65*	*11.0*	0	*65*	*12.0*	0	*70*
D_2_O ^¥^		–	s-i	6.0	–	s-i	6.0	–	s-i	6.0	–

Notes: Bold parameters were varied during fitting, parameters in *italic* were dependent on values of related bold parameters, and all other parameters were fixed. For derivation of fixed parameters see legend of Table 1. Scattering background was set to 5 × 10^−^^5^ for 20 °C, and 8 × 10^−^^5^ for 38 °C. Δ*Q*/*Q* was set to 5% for pH = 5 and 20% for pH = 9. ^¥^ The *SLD_D2O_* deviated from its literature value due to contamination with H_2_O at a volume fraction of 0.053.

### 2.3. Data Modeling

**Figure 4:**
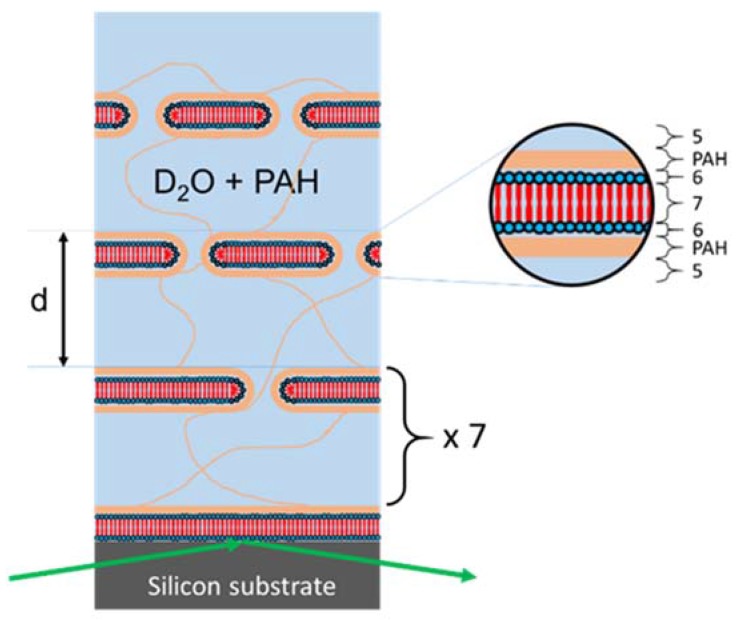
Model of the solid-supported oligolamellar DMPC linings against polymer solution (3 mg/mL PAH in D_2_O) applied for fitting. The neutron beam (green arrows) probes the interfacial coating through the silicon fronting. The labels in the enlargement refer to respective layer numbers in Table 1 (Table 2). PAH adlayer = layer No. 4.

All reflectivity curves were fitted by applying Parratt’s approach of calculating the reflectivity of stratified media employing the Motofit software package [21]. The model of the oligolamellar lipid coatings (see Figure 4) was based on the model utilized by Kreuzer *et al.* [13]. It consisted - from bottom to top - of a Si fronting, a native SiO_2_ layer, a first layer of DMPC heads, heads_a_, including hydration water, tails_a_ (2× tails of DMPC), heads_a_, and a PAH adlayer bound to the outer headgroup slab of this first, adsorbed DMPC bilayer. This first lipid membrane was the anchor of the free floating part of the oligolamellar stack which reads as a nine-fold repetition of water interlayer, PAH adlayer, heads, tails, heads, PAH adlayer against the bulk aqueous solution. The SLD of the water interlayer and that of the bulk solution were always kept identical. Deviations of the SLD of the bulk solution from the theoretical value of D_2_O were due to contamination with H_2_O and PAH content. The best-fit parameters are listed in Table 1 (Table 2) for sample A (sample B).

We kept the fit model as strict as possible. The structural parameters (*d*, *SLD*) of Si, SiO_2_, DMPC heads and DMPC tails were taken from literature and adopted to the current situation with respect to temperature and solvent *SLD* but not varied otherwise (see legend of Table 1). The same held for *SLD_PAH_*. In addition, the adsorbed DMPC membrane (*N* = 1) and the remaining pile of nine lipid bilayers were kept identical as were the water interlayers and the PAH adlayers for any given condition in *T* and *pH*. We varied exclusively the thickness of the water interlayers, the thickness of the PAH adlayers, and the solvent fraction of adsorbed DMPC bilayer, free floating DMPC bilayers and PAH adlayers. In total, five adjustable parameters sufficed in describing the measured systems at all conditions in a satisfactory manner.

### 2.4. (Vertical) Structure

The conducted measurements reveal that, when in contact with the dilute polyelectrolyte solutions, the oligolamellar lipid membranes remain highly ordered, uniform and parallel to the substrate surface, but separated from each other by large water interlayers. An increase in membrane-to-membrane distance, *i.e.*, a larger *d*-spacing is expected for the oligolamellar stacks of lipid membranes in liquid water: The membrane systems fully hydrate and develop a water layer between the opposing membranes [22,23]. According to literature, a fully hydrated stack of DMPC lipid membranes at 20 °C shows a *d*-spacing between 64 and 66 Å [24,25]. This is in strong contrast to the systems presented here which were incubated in solutions of PAH in D_2_O with a measured *d*-spacing of 257 ± 2 Å for system A and 239 ± 1 Å for system B. The water interlayer of DMPC membranes in water is 11.6 ± 1 Å at 10 °C [26] and 18.5 Å at 30 °C [19]. In our systems the water interlayer at 20 °C is on average by a factor of 16 larger (*cf.* Table 1 and Table 2). This drastic swelling introduced by PAH is further corroborated in an independent study [27]. 

The DMPC bilayer membranes are not perfect. With the exception of the very first DMPC membrane, directly adsorbed to the silicon substrate, they host a substantial amount of water (potentially including PAH at bulk concentration) within the individual bilayer leaflets varying between 45% (sample A) and 21% (sample B) and further changing with treatment (*T*, *t*, and pH), see Table 1, Table 2 and Figure 4. Such high amounts of solvent within solid-supported lipid membranes are not uncommon [28,29,30]. The solvent partitions the bilayers into patches on a length scale smaller than the coherence length of the neutron beam. This allows for simulation on basis of a binary composition of the strata consisting of material *m* and solvent *sol* with *SLD_stratum_* = (1 − *ϕ*) ⋅ *SLD_m_* + *ϕ* ⋅ *SLD_sol_*. The coherence length of the neutron beam in our case was of the order of 5–50 microns [31]. We found the individual headgroup slabs decorated with PAH adlayers at a polymer concentration of typically 40% by volume (except for sample B at pH 9 where it was 30%). The thickness of the PAH adlayers was between 7 and 12.4 Å dependent on *T*, *t*, and pH. The enrichment of PAH in the adlayers was thus 200 times above bulk concentration (0.2% PAH by volume).

**Figure 5:**
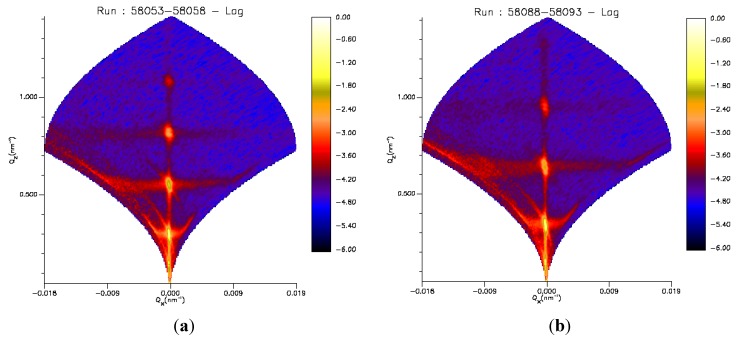
Neutron scattering maps of sample B at the solid–liquid interface incubated with PAH-15 in D_2_O at 20 °C (**a**) and 38 °C (**b**) with specular Bragg peaks up to fourth order on the *Q_z_* axis at *Q_x_* = 0, *i.e.*, perpendicular to the interface. Diffuse bands of scattered intensity (Bragg sheets) intercepting the Bragg peaks and extending to finite *Q_x_* at constant *Q_z_*, *i.e.*, parallel to the interface, are also visible.

Figure 5 shows the 2D scattering maps of sample B at a solution pH of 5 at 20 and 38 °C, respectively. Similar maps are obtained for sample A. The intense Bragg sheets in Q_*x*_ direction are a result of the high order of the substrate-bound lipid membranes. The Bragg sheets can originate from conformal thermal fluctuations or correlated layer distortions around the lamellar defects [32]. In both cases, their existence proves a strong coupling of the individual DMPC bilayers across the stack on the micron scale.

### 2.5. Temperature and Stability

The main phase transition temperature *T_m_* of the lipid molecules from their gel state, $P_{ß}^{\prime }$, to their liquid state, *L_α_*, lies around 24 °C [15,24]. Heating samples A and B from 20 to 38 °C, *i.e.*, from a temperature below *T_m_* to a temperature above *T_m_*, shifted the Bragg peak positions to higher *Q*-values (smaller *d*-spacing). Figure 2 (Figure 3) shows the temperature dependent reflectivity of sample A (sample B). The measurements state lamellar ordering in all cases. Upon changing to 38 °C, the *d*-spacing of sample A shrinks by 44 Å and that of sample B by 37 Å (*cf.* Table 1 and Table 2). In the course of the main phase transition, the lipid chains “melt” from their ordered all-trans configuration to their disordered state with increasing number of gauche conformations (kinks) [33]. Concomitantly, in the bulk lyotropic reference system the *d*-spacing reduces from 65 Å at 20 °C to 61 Å at 38 °C, *i.e.*, by 4 Å [24]. In the pure DMPC/water system, an unbinding process occurs which for oligolamellar lipid coatings results in the loss of the substrate-bound lipid bilayers [13,34]. Our results on the DMPC/(water+PAH) systems—on the opposite—show: (i) that the lipid linings remain stable on the silicon substrate at elevated temperature, and (ii) that the observed Δ*d* of −44 Å for sample A and −37 Å for sample B cannot be explained by the change of the phase state of the lipid bilayers. The analyses of the measured NR curves (Figure 2, Figure 3, Table 1 and Table 2) reveal that the variation in thickness of the water interlayer, *d_w_*, is the main cause of the detected effect. It reduces by 38 Å for sample A and by 32 Å for sample B. In line with concomitant reductions in DMPC head and tail regions of 2 ×(−3) Å + (−5) Å = −11 Å (samples A and B), it overcompensates a slight increase in PAH adlayer thickness of 2 ×2.9 Å ≈ 6 Å (sample A) and 2 ×2.3 Å ≈ 5 Å (sample B).

The temperature effect is reversible as Figure 2 reveals. Upon cooling sample A from 38 to 20 °C, the water interlayer increased in thickness, as did the lipid heads and tails slabs while the PAH adlayers decreased in thickness again (see Table 2).

### 2.6. Time

The swelling of an oligolamellar DMPC coating upon incubation with HA-769 (*M_w_* = 769 kDa) in D_2_O is reported to be a very slow process, the lipid lining changed continuously in the first four days. When re-measured after 43 days a stable and well-defined structure was found [13]. In contrast, the swelling induced by PAH seems much faster: After four days of incubation in the solution of PAH-58, sample A was fully equilibrated and in its final state. The observations suggest a dependence of equilibration time on molecular weight. The NR curve of sample A at 38 °C (see Figure 2) was recorded five days after the first measurement at 20 °C. It shows four well-pronounced Bragg peaks. After heating to 38 °C, we followed the cooling from 38 to 20 °C with a time resolution of 8 min per NR measurement in a limited *Q*-region from 0.024 to 0.033 Å^−1^. We found, after 8 h at 20 °C, a *d*-spacing of 264 ± 1 Å, which was not yet the final value. With equilibration time, the Bragg peaks sharpened and appeared eventually up to the fifth order. The final measurement, 48 h after the T-jump from 38 to 20 °C, revealed a further increase in *d*-spacing to 270 ± 2 Å (Figure 2, bottom curve). In this latter case, the fit to the NR curve captures all measured features. Therefore, we believe that equilibrium at a given temperature was reached within four days of incubation with PAH-58 and within two days after changes in *T*. In summary, it seems safe to say that the response of the swollen DMPC lining to the *T*-jumps in terms of thickness is fast, *i.e.*, on a time scale of hours, while annealing is slow, *i.e.*, on a time scale of days, but still by a factor of ~4 faster than in the case of HA.

### 2.7. Molecular Weight

The structures of the lipid linings that form upon interaction with the synthetic PAH polyelectrolytes are interesting in the context of the structures found for the same linings upon interaction with the natural occurring and negatively charged polysaccharide HA [13]. In the latter case, the *d*-spacing of the oligolamellar lipid bilayers stack increased from 65 to 247 Å at 38 °C. Table 3 accumulates all findings. With increasing molecular weight at a given temperature also a larger *d*-spacing is established, independent of the type of polyelectrolyte. The molecular weight of HA-769 is by a factor of 13 larger than the molecular weight of PAH-58. The respective *d*-spacings, however, only differ by a factor of 1.2 ± 0.1 at 38 °C and 0.9 ± 0.1 at 20 °C (*i.e.*, before heating). The ratio of PAH molecular weights is 3.9, while the ratio of the respective *d*-spacings found is 1.1 ± 0.1 (before heating). These observations indicate that the molecular weight of the polyelectrolyte utilized to swell the lipid membranes stack only plays a minor role, if at all.

**Table 3:** *d*-spacings of oligolamellar DMPC bilayer stacks in polyelectrolyte solution.

Sample	Solution D_2_O + Polymer (c = 3 mg/mL)	d @ 20 °C (Å) pH = 5 DI_PAH_ = 95%	d @ 38 °C (Å) pH = 5 DI_PAH_ = 95%	d @ 38 °C (Å) pH = 9 DI_PAH_ = 45%	R_E_ (Å) by Equation (3)
Lit1 [20]	–	65 ± 1	63 ± 1	n/a	–
Lit2 [13]	HA-769	234 ± 1 *	247 ± 1	n/a	1637
A	PAH-58	257 ± 2 ^a^	213 ± 2	n/a	388
		270 ± 2 ^b^			
B	PAH-15	239 ± 1	202 ± 1	76 ± 1	172

Notes: * not published; n/a = not available, ^a^ before heating; ^b^ after heating.

### 2.8. Charge

PAH has a pKa of approximately 8.8 [35]. At a solution pH of 5, the polyelectrolyte chains are ionized to ~95% (positively charged). In this state, the *d*-spacing measured for sample B at 38 °C (see Figure 3, middle curve) was d = 202 ± 1 Å. The situation changed drastically after rendering the solution pH to 9 (by adding 20 µL of 30 % NaOD in D_2_O to the liquid phase of the sample cell). At this pH, only around 50% of the PAH units stay charged [35] with direct impact on the structure of the lipid coating as Figure 3, bottom curve, reveals. Instead of four, only one broad Bragg peak remained at *Q* = 0.083 Å^−1^. The corresponding *d*-spacing is 75.8 ± 0.2 Å. Subsequent measurements showed that the system was stable in this condition within 12 h until the end of the beamtime. From the experiment it is evident that the surface charges, installed with the PAH adlayers on the DMPC bilayers, control the separation of adjacent membranes. This finding is in-line with earlier findings on the effect of electrostatic repulsion caused by Ca^2+^ ions adsorbed to opposing DPPC membranes reported by Ohshima and Mitsui [36]. Upon de-ionization of the PAH molecules at pH 9, the electrostatic repulsion diminishes and the attractive membrane interaction forces dominate the membrane-to-membrane distance. The resulting *d*-spacing is by only 15 Å larger than that of fully hydrated stacks of DMPC lipid membranes in pure water at the same temperature [24]. The difference is twice the thickness of the PAH-adlayer in this state (see Table 3). That is to say, the DMPC bilayers stack incubated with PAH collapses to its maximum possible extent.

### 2.9. Balance of Forces

With the presumption that the individual contributions to the balance of forces can be separated, the disjoining pressure of the lipid membranes as a function of distance *d* will read as

(1) $$\Pi \left(d\right)=\Pi_{vw}\left(d\right)+\Pi_{es}\left(d\right)+\Pi_{st}\left(d\right)+\Pi_{hr}\left(d\right)$$

The electrostatic forces are represented by Π_*es*_(*d*), the steric forces of the confined polymer by Π_*st*_(*d*), and the hydration repulsion forces by Π_*hr*_(*d*). In sum, these forces compete against the attractive van der Waals forces resembled in Π_*vw*_(*d*) = −*A_h_*/(6*πd*^3^) with the effective Hamaker constant *A_h_* [37,38].

The electrostatic forces between the charged bilayers scale as

(2) $$\Pi_{es}\left(d\right)\;\sim \;B\cdot \text{exp}\left(-\kappa d\right)$$

where *B* = *B*(*c*_0_, *σ_s_*, *T*), and is a function of the bulk concentration of the counterions, *c*_0_, the surface charge density, *σ_s_*, and temperature, *T* [39]. Cowley *et al.* report the repulsive forces between charged lipid bilayers to be the dominant contribution at large interbilayer separations *d_w_* [40]. In concordance with the findings for the zwitterionic DPPC bilayer, membranes charged by adsorbed Ca^2+^ ions [36].

The steric forces entering Π_*st*_(*d*) are brought about by the polyelectrolyte molecules, which in this work were dissolved in pure D_2_O with no extra salt added. The bulk concentration of monomers was 0.032 mol/L. The Cl(-) counterions coming with the dissolved PAH(+) would screen the charges on the polymer molecules at room temperature on the length scale of $\kappa^{-1}=3.04\;Å\;/\sqrt{I\left(mol/L\right)}$, where *I* is the ionic strength of the solution [41]. The resultant screening length *κ*^−1^ = 17 Å in our case. This length is larger than the bare persistence length *l*_0_ ≅ *b* = 5 Å, which is the bond length or diameter of an allylamine hydrochloride monomer unit, AH. On the other hand, *κ*^−1^ ≪ *b* ⋅ *N*, where *b* ⋅ *N* is the contour length of the polymer molecule made up by the *N* monomers. In line with Odijk, Skolnick and Fixman (in Netz and Andelman, 2002 [42]), we define an electrostatic persistence length *l*_OSF_ = *l*_B_*τ*^2^/4*κ*^2^ with the Bjerrum length *l*_B_ of water of 7 Å and the linear charge density along the polymer backbone of *τ* = *f*/*b* = 0.19 Å^−1^ at the given degree of ionization, *f* = 0.95. Consequently, the effective persistence length would read as *l*_eff_ ≅ *l*_0_ + *l*_OSF_ and is 5 Å + 18 Å = 23 Å. Hence, *l*_eff_ ≅ *κ*^−1^. As the contour length *L* ≫ *l*_eff_, we treat the polymer coil - as outlined in Netz and Andelman 2002 - as a solution of separate polymer pieces of length *l*_eff_ and diameter *d_p_* and describe their interactions by their respective second virial coefficient $v_{2}\;\sim \;l_{\text{eff}}^{2}d_{p}$. For our charged polymer, at very low ionic strength, the effective rod diameter is *d_p_* ~ *κ*^−1^. Under the experimental circumstances, the end-to-end distance *R_E_* was (see Equation (25) in R. Netz and D. Andelman, 2002 [42]):

(3) $$R_{E}\sim {\left(\frac{v_{2}}{l_{\text{eff}}}\right)}^{1/5}L^{3/5}\sim {\left(\kappa^{-1}\right)}^{2/5}{\left(b\cdot N\right)}^{3/5}$$

**Figure 6:**
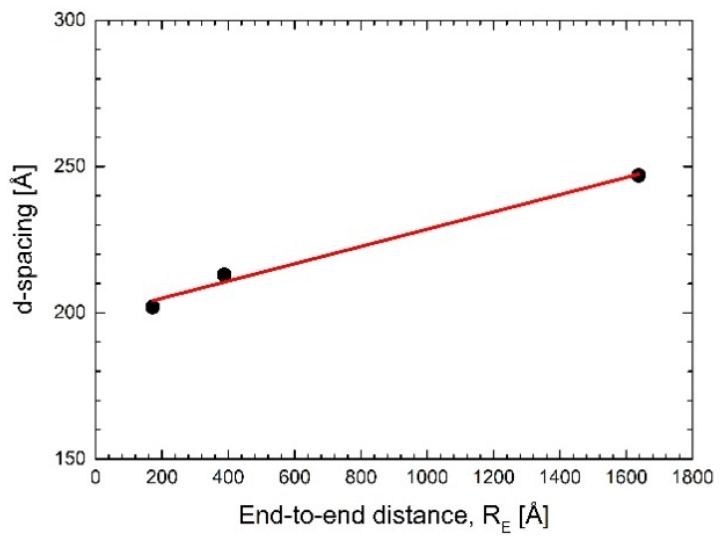
Measured *d*-spacing of the oligolamellar DMPC linings incubated in polyelectrolyte solutions at a concentration of 3 mg/mL polymer in D_2_O at 38 °C plotted *versus* the end-to-end distance of the respective polymer (*cf.* Table 3). The solid line is a linear fit to the data according to *d* = *mR_E_* + *d*_0_ with *m* = 0.029 ± 0.003 and *d*_0_=199 ± 3 Å.

In the case of PAH-15, *R_E_* < *d_w_*, while for PAH-58, *R_E_* > *d_w_*, and for HA-769, *R_E_* ≫ *d_w_* (see Table 1, Table 2 and Table 3 and ref. [13]). Hence, with increasing molecular weight, the polymer molecules entrapped within the water interlayer between adjacent lipid bilayer membranes would experience increasing confinement and thus an increasing entropic penalty. The numbers plotted in Figure 6, however, suggest that the entropy-driven repulsion caused by the confinement does not contribute much to the observed *d*-spacing. In addition, the corresponding analysis with respect to polymer molecular weight shows that the measured net increase in *d*-spacing is only ~0.05 Å/kDa.

Hydration repulsion (*hr*), and thus Π_*hr*_(*d*), seems negligible in the system investigated here. Schneck and coworkers showed in a molecular dynamics study on interacting DPPC bilayers that the net interaction between the lipid membranes due to *hr* vanishes for water interlayers *d_w_* larger than 20 Å [43]. From the work of Smirnova *et al.* on interbilayer repulsion forces between tension-free DOPC bilayers, *cf.* their Figure 6, it is evident that the disjoining pressure in such lipid systems is vanishing for an interwater layer thickness of *d_w_* ≥ 66 Å [44]. The findings are corroborated by earlier work, which reports *d_w_* = 26.5 Å, *i.e.*, much smaller than *d_w_* measured here, for plain DMPC lamellar structures in excess water at 27 °C [38].

The dominant force seems the electrostatic repulsion between the charged lipid membranes after polymer adsorption. This adsorption is based on the dipole–monopole interaction of the zwitterionic lipid headgroups and the charged units of the polymer, and results in a polymer ad-layer with thickness *d*_PAH_ < *l*_eff_ in our case (see Table 1 and Table 2). This is also true in the HA-case with *d*_HA_ = 22 Å [13] and *l*_eff_ ≅ 34 Å. From the experiments we have no direct measure of the resultant surface charge density *σ_s_* of the adlayer entering the prefactor *B* in Equation (2). If *B* was independent of *T*, then the ratio *d*_*w*1_/*d*_*w*2_ would scale as $\sqrt{\varepsilon_{1}T_{1}/\varepsilon_{2}T_{2}}$, where *ε* is the temperature dependent relative permittivity of water and *T* is given in Kelvin. In the temperature range investigated, $\sqrt{\varepsilon_{1}T_{1}/\varepsilon_{2}T_{2}}$ = $\sqrt{80.1\cdot 293/73.8\cdot 311}$ = 1.01 and is practically constant. The experimental ratio *d*_*w*1_/*d*_*w*2_, however, varies substantially (Table 1 and Table 2). It is 1.26 ± 0.04 for sample A and 1.21 ± 0.04 for sample B, and underlines that *B* = *B*(*T*) and determines the equilibrium distance between the membranes.

With $\tau \sqrt{l_{0}l_{B}}$ = 0.19 × (5 × 7)^1/2^ = 1.12 > 1, we are in the so-called persistent regime of the polyelectrolyte molecule [42]. We therefore assume condensation of the counterions in the close vicinity of the polymer chain. As the chain in this regime is locally rather straight, globally the additional influence of the osmotic pressure of the condensed counterions on the chain structure might not be profound [42].

In summary, it is the electrostatic repulsion of the charged lipid bilayers through polyelectrolyte adsorption, which governs the *d*-spacing of the oligo-stacks at the solution-solid interface. Switching off this force by changing pH from 5 to 9 immediately results in a *d*-spacing comparable to that of simple DMPC oligo- and multilamellar stacks at the aqueous–solid interface.

## 3. Experimental Section

### 3.1. Chemicals

Poly-(allylamine hydrochloride) (PAH) with average molecular weights of *M_w_* = 58 kDa (PAH-58) for sample A and *M_w_* = 15 kDa (PAH-15) for sample B were purchased from Aldrich and used as received. 1,2-Dimyristoyl-sn-glycero-3-phosphocholine (DMPC) lipid was ordered from Avanti Polar Lipids, Inc., Alabaster, Alabama, US, and used without further purification. Ultrapure water was obtained by using a Milli-Q purification system (resistance >18.2 MOhm·cm) and D_2_O was purchased from Aldrich (purity ≥ 99.9 atom % D). Ethanol, p.a. grade, was from Sigma-Aldrich. Chloroform, uvasole grade, was purchased from Merck.

The end-to-end distance, *R_E_*, of a polymer in solution was calculated using Equation (3) with the size *b* of an allylamine hydrochloride (AH) monomer of 5.04 Å, the molecular weight of the AH-monomer of 0.093 kDa and the degree of polymerization, *N* = *M*_w,PAH_/*M*_w,AH_. The respective numbers for HA are *b* = 10.62 Å, and *M_w,HA−monomer_* = 0.378 kDa.

### 3.2. Sample Preparation

Block-shaped silicon substrates (A and B) with dimensions of 80 mm length ×50 mm width ×15 mm thickness and one polished surface (80 mm ×50 mm; rms-roughness ≤ 6 Å) were utilized. They were polished and supplied by Siliziumbearbeitung Holm (Tann/Ndb., Germany). Both substrates (A and B) were cleaned for 1 hour in an ethanol bath and subsequently rinsed for 10 min in ultrapure water. Oligolamellar lipid coatings were prepared on the pre-cleaned substrates by spin-coating from 10 mg/mL lipid solutions in chloroform at a speed of 4000 rpm, using a spin-coater (Model 6708D, Specialty Coating Systems, Inc., Indianapolis, IN, USA) and following the procedure described by Mennicke and Salditt [16]. Liquid phases were solutions of 58 kDa PAH (PAH-58) in D_2_O for sample A and 15 kDa PAH (PAH-15) in D_2_O for sample B. A concentration of 3 mg PAH/mL D_2_O was chosen in both cases in order to compare the results with previous measurements [13].

### 3.3. X-Ray Reflectometry

X-ray reflectivity experiments were performed at ambient humidity at room temperature on a triple axis diffractometer using Cu Kα (λ = 1.54 Å) radiation. The resolution of the instrument was set to *δQ_z_* = 0.003 Å^−1^. Details on the instrument and its operational mode can be found elsewhere [45].

### 3.4. Neutron Reflectometry

For *in-situ* neutron reflectivity measurements, a home-built flow cell especially designed for experiments at the solid–liquid interface was used [46]. Measurements were performed at the V6 instrument at BER II, Helmholtz-Zentrum Berlin für Materialien und Energie GmbH. Sample temperature was controlled by a water thermostat and measured with a Pt100 thermocouple placed underneath the liquid subphase. A neutron wavelength of λ = 4.66 Å was selected by a pyrolytic graphite crystal PG 002 located in the incident white beam. The angular resolution was set by a slit system on the incident side resulting in scattering vector resolution *δQ* of 0.001 Å^−1^ for *Q* < 0.0518 Å^−1^, and 0.002 Å^−1^ otherwise. The measurements were performed in *θ*/2*θ* geometry. The scattered neutrons were recorded with a position sensitive detector (PSD).

### 3.5. Reflectometry—Data Analysis

The specular reflectivity curves (at *Q_x_* = 0), measured by X-rays or neutrons, were normalized to time and initial beam intensity and displayed against the momentum transfer *Q* = *Q_z_* = (4*π*/*λ*) ⋅ *sin θ* perpendicular to the sample surface, with *θ* being the reflection angle. The reflectivity curves showed interference fringes arising from X-rays or neutrons that were reflected at the interface “substrate/lipid coating” and at the interface “lipid coating/environment”. These so-called Kiessig-fringes represent the total film thickness, *t*, of the oligolamellar lipid coating in reciprocal space. By applying Bragg’s law, *t* was read out from the distance *ΔQ_K_* between adjacent Kiessig maxima according to *t* = 2*π*/*ΔQ_K_*. In addition, the measured reflectivity curves showed characteristic Bragg peaks at positions *Q_B,n_* up to *n*-th order, which evolved from the interference of X-rays or neutrons that were reflected from the one dimensional grating composed of individual lipid lamellae within the oligolamellar stack. The latter peaks contain information on the spacing, *d*, of individual lipid lamellae within the coating. The lamellar spacing *d* was extracted from the measurements by applying Bragg’s law as *d* = 2*π* ⋅ *n*/*Q_B,n_*. The number *N* of lamellae within a stack is given as *N* = *t*/*d*. The appearance of Kiessig oscillations made it possible to calculate the total film thickness of *t* and the total number *N* of lipid membranes.

## 4. Conclusions

After incubating silicon-supported zwitterionic DMPC bilayer membranes in a solution of the positively charged polyelectrolyte PAH in D_2_O (3 mg/mL) at pH 5, the lipid linings swell heavily. The *d*-spacing increases by a factor of ~4 when compared to the reference system against pure D_2_O. Upon increasing temperature from 20 to 38 °C, *i.e.*, from *T* < *T_m_* to *T* > *T_m_*, the *d*-spacings decrease only marginally. When increasing solution pH from 5 to 9, the lipid membrane system B, investigated in this respect, returns to almost the expected *d*-spacing of the reference system in pure D_2_O. We identify electrostatic interactions introduced by the adsorbed PAH to be responsible for the drastic swelling of the lipid linings. The DMPC membrane stacks do not detach from their solid support at *T* > *T_m_*, as opposed to the behaviour of the reference system. We hold steric interactions, also introduced by the PAH molecules, liable for that stabilizing effect. The clear identification of the effects of molecular weight and pH is beyond the scope of this study and requires further work.
